# Bone Morphogenetic Protein 15 in the Pro-Mature Complex Form Enhances Bovine Oocyte Developmental Competence

**DOI:** 10.1371/journal.pone.0103563

**Published:** 2014-07-24

**Authors:** Jaqueline Sudiman, Melanie L. Sutton-McDowall, Lesley J. Ritter, Melissa A. White, David G. Mottershead, Jeremy G. Thompson, Robert B. Gilchrist

**Affiliations:** 1 Robinson Research Institute, Research Centre for Reproductive Health, and School of Paediatrics and Reproductive Health, Discipline of Obstetrics and Gynaecology, Medical School, University of Adelaide, Adelaide, South Australia, Australia; 2 Discipline of Obstetrics & Gynaecology, School of Women's & Children's Health, University of New South Wales, Sydney, New South Wales, Australia; Justus-Liebig-Universität, Germany

## Abstract

Developmental competence of *in vitro* matured (IVM) oocytes needs to be improved and this can potentially be achieved by adding recombinant bone morphogenetic protein 15 (BMP15) or growth differentiation factor (GDF9) to IVM. The aim of this study was to determine the effect of a purified pro-mature complex form of recombinant human BMP15 versus the commercially available bioactive forms of BMP15 and GDF9 (both isolated mature regions) during IVM on bovine embryo development and metabolic activity. Bovine cumulus oocyte complexes (COCs) were matured *in vitro* in control medium or treated with 100 ng/ml pro-mature BMP15, mature BMP15 or mature GDF9 +/− FSH. Metabolic measures of glucose uptake and lactate production from COCs and autofluorescence of NAD(P)H, FAD and GSH were measured in oocytes after IVM. Following *in vitro* fertilisation and embryo culture, day 8 blastocysts were stained for cell numbers. COCs matured in medium +/− FSH containing pro-mature BMP15 displayed significantly improved blastocyst development (57.7±3.9%, 43.5±4.2%) compared to controls (43.3±2.4%, 28.9±3.7%) and to mature GDF9+FSH (36.1±3.0%). The mature form of BMP15 produced intermediate levels of blastocyst development; not significantly different to control or pro-mature BMP15 levels. Pro-mature BMP15 increased intra-oocyte NAD(P)H, and reduced glutathione (GSH) levels were increased by both forms of BMP15 in the absence of FSH. Exogenous BMP15 in its pro-mature form during IVM provides a functional source of oocyte-secreted factors to improve bovine blastocyst development. This form of BMP15 may prove useful for improving cattle and human artificial reproductive technologies.

## Introduction

The oocyte and the somatic cells surrounding the oocyte (cumulus cells in antral follicles) communicate via a complex bi-directional signalling axis mediated via paracrine and gap junctional means [1]. This communication is important for transporting molecules such as growth factors, cyclic nucleotides, amino acids and other small regulatory molecules from cumulus cells into oocytes, and vice versa, in order to sustain cumulus cell health and oocyte development [1]–[4]. Oocyte-secreted growth factors (OSFs) act on cumulus cells to regulate multiple functions, including; differentiation of the cumulus cell lineage [5], the proliferation and expansion of cumulus cells [6]–[9], prevention of luteinisation [5], [10] and apoptosis [11] and regulation of cumulus cell metabolism [12]–[14]. Healthy cumulus cells are vitally important for the development of oocytes through the provision of substrates and regulatory molecules, required for the oocyte to undergo appropriate developmental programming in order to support early embryo development [15].

Growth differentiation factor 9 (GDF9) and bone morphogenetic protein 15 (BMP15) are two major and well-known OSFs, required for follicular development and ovulation [16]–[19]. The function and expression of both proteins differs markedly between species [20]. In polyovular animals, oocyte expression of GDF9 is notably higher than BMP15, suggesting a minor role for BMP15 in the regulation of their reproduction. Consistent with this notion, homozygous mutant GDF9 mice are sterile due to a block in follicular development beyond the primary follicle stage [16], whereas BMP15 homozygous mutant mice demonstrate only a mild reduction in fertility [21]. In contrast, in monoovulatory animals, the ratio of oocyte GDF9 to BMP15 is nearly equal [20], reflecting the importance of both of these growth factors in follicle development in these species. Sheep homozyous for certain naturally occuring mutations in either GDF9 or BMP15 are sterile [17], [18]. Interestingly, sheep heterozygous for these mutant forms of GDF9 or BMP15 have an increased ovulation rate and incidence of mutiple pregnancies due to increased LH sensitivity in secondary follicles that leads to a rise in the number of antral follicles [17], [18], [22]. Furthermore, in humans, rare mutations and genetic variance of GDF9 and BMP15 are associated with polycystic ovary syndrome [23], dizygotic twinning [24] and premature ovarian failure [25]–[27].

Like other members of the TGFβ superfamily, the pro-proteins (un-processed proteins) of GDF9 and BMP15 are composed of a pro-region and a mature region [28]. The pro-regions of these proteins can only be dissociated from the corresponding mature regions after processing at their protease processing site, after which they form a pro-mature complex held together by non-covalent interactions [29]. There still remains considerable uncertainty exactly which forms of GDF9 and BMP15 are secreted by the oocyte to act on the surrounding cumulus cells, and whether the form of these proteins produced by oocytes matured *in vitro* is similar to what may occur *in vivo*. However, a recent study has shown that in vitro, but not in vivo, matured mouse COCs are deficient in the mature domain of BMP15 [30]. Moreover, sheep follicular fluid contains GDF9 and BMP15 proteins only in the unprocessed pro-protein form [31], whereas sheep oocytes secrete the mature domains of GDF9 and BMP15 during IVM [32]. *In vitro* matured mouse oocytes secrete GDF9 as a mixture of the pro-protein and mature domain [33], whilst *in vitro* matured rat oocytes secrete mature domain GDF9 only [32]. Commercially available GDF9 and BMP15 proteins from the one supplier of these peptides (R&D Systems) contain only the mature regions of these proteins; however, our novel BMP15 contains both pro and mature regions, as a processed pro-mature complex. Consistent with the role of pro-regions of other TGFβ superfamily members, it has now been demonstrated that the pro-regions of GDF9 interact with cumulus cells [34]. Hence, we hypothesised that the presence of the pro-region is important to retain the full function of BMP15 or GDF9 and may affect oocyte developmental competence.

Our group has previously shown that addition of partially purified GDF9 or BMP15, which contains a mixture of proteins including the pro-mature complex form, to *in vitro* maturation (IVM) medium improves mouse and cattle blastocyst development [35]–[37]. Moreover, addition of a purified BMP15 pro-mature complex to IVM medium increases bovine embryo development [14], [38]. By contrast, we have recently shown that addition of the isolated mature homodomains of GDF9 and/or BMP15 (from R&D Systems) has no effect on mouse IVM [39]. No studies to date have directly compared the effect of the form of GDF9 or BMP15 added during IVM on embryo development. In this study, we examined the effect of the mature (R&D Systems) and pro-mature (in-house) forms of human BMP15, as well as the mature form of mouse GDF9 (R&D Systems), on bovine oocyte developmental competence following IVM. We examined the metabolic activity of COCs, including glucose uptake and lactate production, as well as mitochondrial and antioxidant activity that occurred during the maturation of bovine oocytes.

## Materials and Methods

Unless otherwise specified, all chemicals and reagents were purchased from Sigma (St Louis, MO).

### Collection of cells

Bovine ovaries were collected from a local abattoir (T&R Pastoral, Murray Bridge, South Australia) and transported to the laboratory in 30–35°C saline. Cumulus-oocyte complexes (COCs) and mural granulosa cells (GCs) were aspirated from 3–8 mm antral follicles using an 18-gauge needle and a 10 ml syringe. The follicular fluid containing COCs was transferred into a 10 ml Falcon tube at 39°C. The follicular aspirate was allowed to sediment and the cellular pellet was washed with handling medium (HEPES-buffered tissue cultured medium-199; TCM-199, MP Biomedicals, Solon, OH, USA) supplemented with 4 mg/ml fatty-acid-free bovine serum albumin (FAF-BSA; ICPbio Ltd, Auckland, NZ). Intact COCs with compact cumulus cells, >3 cell layers and with an evenly pigmented oocyte cytoplasm, were selected under a dissecting microscope and washed twice in handling medium.

### Granulosa cell thymidine assay

To confirm that the forms of recombinant GDF9 and BMP15 used in this study were bioactive on bovine ovarian cells, a standard granulosa cell tritiated thymidine incorporation bioassay was performed, as previously described [6], [40]. In brief, GCs were collected from 3–8 mm follicles. The GCs were washed twice in protein-free culture medium (bicarbonate-buffered TCM-199). Cells were added to the wells of 96-well plates (Falcon) at a final concentration of 50×10^4^ cells/ml, and cultured at 37°C, 96% humidity in 5% CO_2_ in air for 18 hours, followed by a further 6 hour pulse of 15.4 kBq tritiated thymidine ([^3^H]-thymidine, MP Biomedicals) under the same conditions. Following culture, GCs were harvested and incorporated [^3^H]-thymidine was quantified using a scintillation counter as an indicator of GC DNA synthesis. Each treatment was performed in duplicate and the assay was repeated five times.

### Sources of BMP15 and GDF9 and treatment of cumulus-oocyte complexes

COCs were matured in base IVM medium [bicarbonate-buffered TCM 199+4 mg/ml FAF BSA +/−0.1 IU/ml FSH (Puregon, Organon, Oss, Netherlands)] in a 4-well Nunc dish (Nunclon, Denmark) and cultured for 23 hours at 39°C with 5% CO_2_ in humidified air. Recombinant mouse mature region GDF9 (Cat No: 739-G9) and human mature region BMP15 (Cat No: 5096-BM) were purchased from R&D Systems (Minnaepolis, MN, USA).

**Figure 1:**
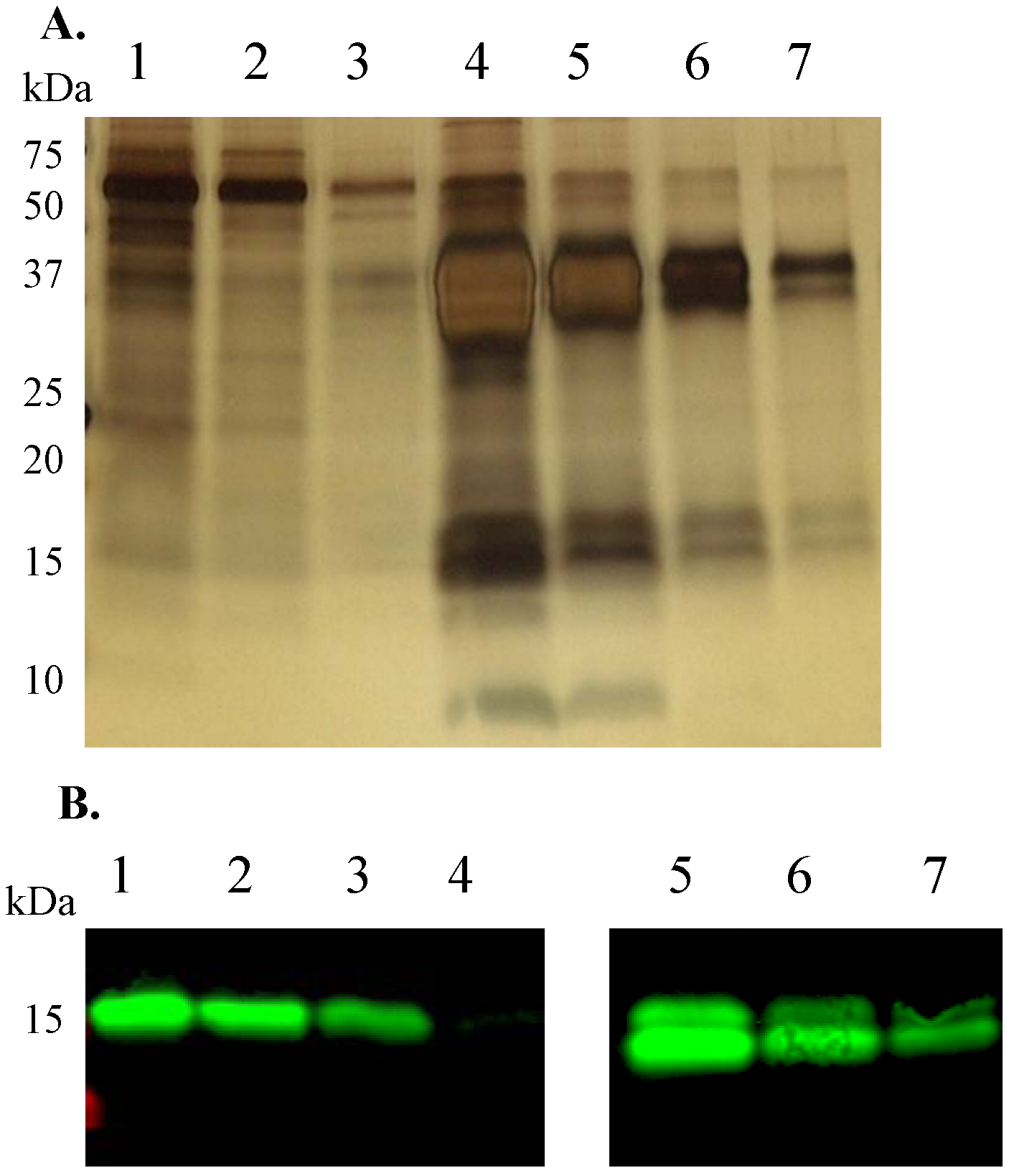
Purification and quantification of pro-mature BMP15. **A.** Silver stained SDS-polyacrylamide gel of BMP15 samples. The pro-region appeared as two ∼40 KDa bands and the mature region as a ∼16 KDa band and as a ∼17 KDa band that has been reported to be O-linked glycosylated [68]. Lane 1: flow through. Lanes 2–3: wash 1–2. Lanes 4–7: eluted fractions 1–4. **B.** The processed mature region of pro-mature BMP15 was quantified by Western blotting [mab28 monoclonal antibody [42]] using the mature region of hBMP15 (R&D Systems) as a standard. Lanes 1–4: mature region of BMP15 (R&D Systems); 200, 100, 50 and 10 ng, respectively. Lanes 5–7: decreasing doses of the purified BMP15 pro-mature complex.

The recombinant human pro-mature complex of BMP15 was produced from a stable human embryonic kidney (HEK)-293T cell line generated in our laboratory. Briefly, an expression cassette encoding the human BMP15 DNA sequence was synthesized (Genscript USA, Inc., Piscataway, NJ) incorporating the rat serum albumin signal sequence at the 5′ end followed by a His8 tag and a Strep II epitope tag at the N-terminus of the BMP15 pro-region. This expression cassette was transferred to the pEF-IRES expression vector, as previously described [41] and stably transfected HEK-293T cell lines producing human BMP15 were established via puromycin selection. For production of the BMP15 protein, confluent monolayers were transferred to serum-free media (DMEM/F12 +0.1 mg/ml BSA +125 IU/ml heparin) for a 48 hour collection period. The resultant BMP15 protein was secreted into the serum-free media with a processing efficiency of cleavage of the pro and mature regions of over 90%. The BMP15 containing production media was subjected to immobilized Ni2+ based ion-affinity purification targeting the His-tag as described previously [42]. Samples were tested for the presence of pro-mature BMP15 protein by silver staining (Fig. 1A). The dose of processed mature region was quantified using Western blotting with the mab28 monoclonal antibody [42] and the commercially available mature region of hBMP15 (R&D Systems) as a standard (Fig. 1B).

The effects of graded doses of the purified pro-mature BMP15 were tested at 10, 100 and 200 ng/ml. Four replicate experiments were performed with 30–40 COCs per dose per replicate. The concentration of pro-mature BMP15 was chosen based on this dose-response assessment of embryo development. For all further experiments the treatment media contained pro-mature BMP15, mature BMP15 or mature GDF9 at 100 ng/ml+/−0.1 IU/ml FSH. Six replicate experiments were performed with 30–40 COCs per treatment per replicate.

### In vitro fertilization (IVF) and embryo culture

*In vitro* production of embryos was undertaken using defined, serum-free media (Bovine Vitro series of media, IVF Vet Solutions, Adelaide, Australia), as previously described [37]. Frozen semen was donated by SEMEX Australia from a single bull of proven fertility and was used in all experiments. Briefly, thawed semen was layered over a discontinuous (45%: 90%) Percoll gradient (GE Health Care, Australia) in a 12 ml Falcon tube (Becton Dickinson, Franklin Lakes, NJ, USA) and centrifuged for 20–25 minutes at 271×g. The supernatant was removed and the pellet resuspended in wash medium [VitroWash (IVF Vet Solutions)+4 mg/ml FAF BSA)] and centrifuged for 5 minutes at 56×g. The supernatant was removed and the pellet re-suspended in IVF medium [VitroFert (IVF Vet Solutions)+4 mg/ml FAF BSA+10 IU/ml heparin] with a final concentration of 1×10^6^ spermatozoa/ml. COCs were inseminated in pre-equilibrated drops of IVF medium for 23 h at 39°C in 6% CO_2_ in humidified air. Post-insemination, cumulus cells were removed by manual pipetting with a 1000 µl Gilson pipette. Five presumptive zygotes were cultured in 20 µl drops of pre-equilibrated cleavage medium [VitroCleave (IVF Vet Solutions)+4 mg/ml BSA+1 mM L-carnitin] overlaid with mineral oil at 39°C in 5% O_2_, 6% CO_2_ and balanced with N_2_, for 5 days. On day 5, embryos were transferred into pre-equilibrated blastocyst medium [VitroBlast (IVF Vet Solutions)+4 mg/ml BSA+1 mM L-carnitin] and cultured for another 3 days. Blastocysts were assessed on day 7 and day 8.

### Blastocyst differential staining

Differential staining was performed using a modified method described by [43]. Expanded, hatching and hatched blastocysts were placed into 1% (v/v) Triton X-100 containing 1 mg/ml propium iodide for 20–30 seconds. Blastocysts were washed in absolute ethanol before incubation in 2.5 mg/ml Hoechst 33342 solution for 2 minutes, then mounted in a drop of glycerol in PBS on microscope slides and covered with a cover slip. Embryos were examined under a fluorescence microscope (Nikon, TE 2000-E) at 200× equipped with an ultraviolet filter (excitation, 340–380 nm; emission, 440–480 nm). The inner cell mass (ICM) appeared blue and trophectoderm (TE) nuclei stained pink. 22–38 blastocysts were stained in each treatment group.

### Glucose and lactate measurements

Groups of 10 COCs were matured in 100 µl of IVM medium. After 23 h COC maturation, spent media were analysed for glucose and lactate levels using a Hitachi 912 chemical analyser (F. Hoffmann-La Roche Ltd; Basel, Switzerland). This assay is based on an enzymatic reaction for glucose consumption and enzymatic colorimetric determination for lactate production. Glucose uptake and lactate production are expressed as pmol/COC/h. Twelve replicate experiments were performed with 10 COCs per treatment, per replicate.

### Autofluorescence of NAD(P)H and FAD

NAD(P)H and FAD levels in the oocyte following IVM were measured by autofluorescence using methodologies previously described and validated [44]. Briefly, ten COCs were matured in 100 µl of IVM medium. At 23 h maturation, COCs were denuded and oocytes were transferred into 10 µl drops of handling medium overlaid with mineral oil in glass-bottomed confocal dishes (Cell E&G; Houston, TX, USA). The autofluorescent intensity of FAD was observed using a blue laser (excitation, 473 nm; emission, 490–520 nm), and NAD(P)H using a violet laser (excitation; 405 nm, emission: 420–520 nm) with a confocal microscope (Olympus; Fluoview FV10i). These measurements were calibrated as previously described [14], [45]. The fluorescence from the oocytes was normalized using a fluorescence standard (Inspeck; Molecular Probes). Laser power, gain and pin-hole settings were kept consistent. Four replicate experiments were performed with 10 oocytes per treatment group for each experiment (n = 4).

### Reduced glutathione (GSH)

At 23 h maturation, COCs were denuded and washed in handling medium. Denuded oocytes were incubated for 30 minutes in 12.5 µM monochlorobimane (MCB, Catt No: 69899) and transferred into 10 µl drops of handling medium overlaid with mineral oil in glass-bottomed confocal dishes (Cell E&G; Houston, TX, USA). The fluorescent intensity was observed using a DAPI filter (excitation, 358 nm; emission, 461 nm) with a confocal microscope (Olympus; Fluoview FV10i). Ten COCs were measured per treatment group per replicate and each experiment was replicated 3 times.

### Statistical analysis

All proportional data for embryo development were arcsine transformed prior to analysis using multivariate analysis of variance (ANOVA). Log transformation was performed if the data were not normally distributed, and analysed using one-way ANOVA for blastoycst cell numbers, glucose production and lactate consumption. Autofluorescence of NADP(H), FAD and GSH were analysed by two-way ANOVA and as there was no main effect of FSH (P>0.05), subsequently the form of GDF9/BMP15 was tested within +/− FSH treatment by one-way-ANOVA followed by a least-significant difference (LSD) post hoc test. All analysis was performed with SPSS version 13 (SPSS Inc, Chicago, IL, USA). Differences were considered significant at P<0.05. Data are expressed as mean ± SEM.

## Results

### Bioactivity of different BMP15 and GDF9 forms

**Figure 2:**
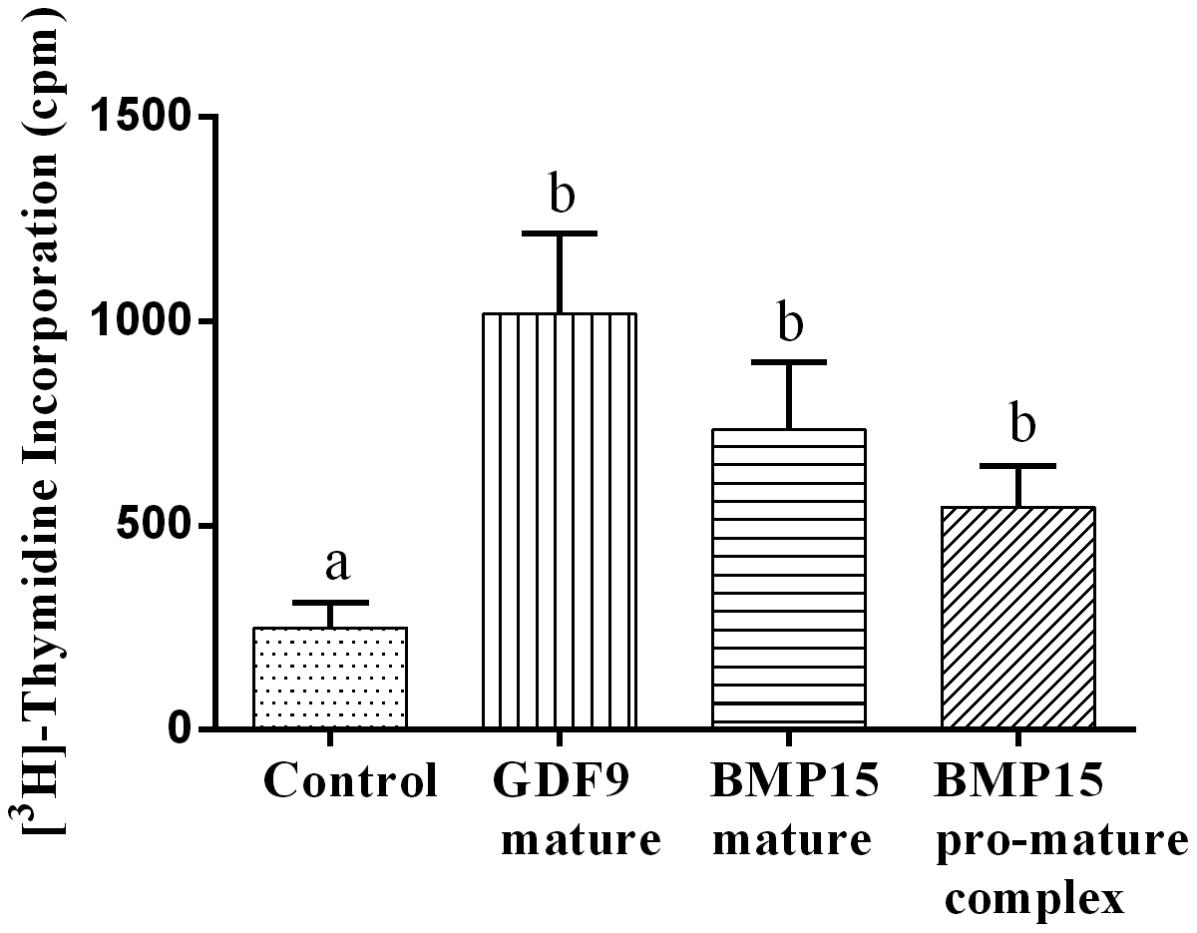
Bioactivity of different BMP15 and GDF9 forms. Granulosa cell tritiated thymidine incorporation following exposure to mature GDF9, mature BMP15 or pro-mature BMP15 at 100 ng/ml. Bars represent mean ± SEM and different lowercase letters indicate a statistically significant difference (P<0.05). Data were derived from 5 independent replicates.

All forms of the three recombinant proteins (pro-mature BMP15, mature BMP15 and mature GDF9) were bioactive on bovine GC, as evidenced by significant stimulation of [^3^H]-thymidine incorporation compared to the control (Fig. 2). The mature region of GDF9 protein tended to cause the highest level of GC [^3^H]-thymidine incorporation and pro-mature BMP15 the least.

### Dose-response of pro-mature BMP15 on oocyte developmental competence

Supplementation of IVM media with pro-mature BMP15 had no effect on subsequent embryo cleavages rates; however, significantly increased blastocyst development on day 8 at each dose examined, compared to control and buffer groups (P<0.05; Table 1). There was no observable dose-response to BMP15 ranging from the lowest dose (10 ng/ml) to the highest dose (200 ng/ml); however, 200 ng/ml produced a high blastocyst rate of 60%; a 1.5-fold higher embryo yield than under control IVM conditions. No adverse effect of elution buffer (PBS/500 mM imidazole buffer) during IVM on either cleavage or blastocyst development was observed (Table 1).

**Table 1:** Effect of graded doses of pro-mature BMP15 during IVM on oocyte developmental competence.

Treatments	No. of oocytes	Cleavage (%)	Blastocyst day 8 per cleaved (%)
Control	151	76.5±3.3	39.9±2.3^a^
Buffer (Imidazole) 500 mM	159	82.2±6.3	40.9±4.6^a^
BMP15 10 ng/ml	151	87.4±5.1	54.0±3.3^b^
BMP15 100 ng/ml	150	83.6±4.9	55.5±6.0^b^
BMP15 200 ng/ml	156	85.8±1.3	59.7±4.4^b^

Values with different superscripts within a column are statistically different (P<0.05). Data are presented as mean ± SEM of 4 experiment replicates.

### Comparison of pro-mature BMP15, mature BMP15 and mature GDF9 during IVM on subsequent embryo development

In the presence of FSH, addition of pro-mature BMP15 to IVM medium significantly increased blastocyst development on day 7 compared to control (Table 2). Irrespective of treatment, there were no significant differences in cleavage rates, day 8 blastocyst rates, inner cell mass, trophectoderm or total cell numbers (Tables 2 and 3). There was a trend towards an improvement in blastocyst development on day 8 from pro-mature BMP15 compared to control, but this was not significant (p = 0.07). The mature form of BMP15 produced intermediate levels of blastocyst development which were not significantly different to pro-mature BMP15 or controls. The mature region of GDF9 did not improve any embryo parameter and blastocyst development on day 7 was significantly lower than both forms of BMP15 (Table 2).

**Table 2:** Effect of supplementing COC during IVM with different forms of BMP15 (mature BMP15 and pro-mature BMP15), mature GDF9 at 100 ng/ml dose, with and without FSH on oocyte developmental competence.

Treatments	FSH	No. of oocytes	Cleavage (%)	Blastocyst day 7 per cleaved (%)	Blastocyst day 8 per cleaved (%)
Control	−	191	76.4±4.8	28.9±3.7^a^	39.3±3.2
GDF9 mature	−	189	85.5±3.1	33.1±9.5a,b	38.4±4.1
BMP15 mature	−	195	84.9±7.3	32.3±4.5a,b	38.5±3.7
BMP15 pro-mature	−	188	81.6±4.3	43.5±4.2^b^	48.7±3.8
Control	+	214	94.2±1.4	43.3±2.4x,y	52.8±3.9
GDF9 mature	+	195	88.8±3.1	36.1±3.0^y^	52.0±4.1
BMP15 mature	+	207	92.1±1.8	50.1±2.6x,z	60.0±3.8
BMP15 pro-mature	+	210	89.1±1.6	57.7±3.9^z^	61.7±.3.5*

Values with different superscript within a column are statistically different (P<0.05);

a,b minus FSH,

x–z plus FSH.

*p = 0.07 relative to control.

Data are presented as means ± SEM of 6 replicate experiments.

**Table 3:** Effect of supplementing COC during IVM with different forms of BMP15 (mature BMP15 and pro-mature BMP15), mature GDF9 at 100 ng/ml dose, with and without FSH on blastocyst quality.

Treatments	FSH	Inner cell mass	Trophectoderm	Total cell number (TCN)	ICM/TCN
Control	−	24.5±1.9	77.3±5.8	101.8±6.3	0.26±0.02
GDF9 mature	−	27.4±2.0	73.8±4.6	101.3±5.2	0.28±0.02
BMP15 mature	−	26.5±1.8	72.1±6.7	98.6±7.9	0.28±0.02
BMP15 pro-mature	−	29.1±1.8	78.6±6.3	107.7±7.1	0.29±0.02
Control	+	30.5±1.6	59.7±3.5	90.2±4.7	0.34±0.01
GDF9 mature	+	28.7±1.9	69.7±5.0	98.4±6.0	0.30±0.01
BMP15 mature	+	31.8±3.0	68.4±4.4	100.2±6.2	0.31±0.02
BMP15 pro-mature	+	34.6±1.8	63.4±2.6	97.9±3.6	0.35±0.01

Data represents the mean ± SEM of 6 replicates. Mean blastocyst cell numbers following differential staining of 22–38 blastocysts.

As expected, in the absence of FSH, all embryo development and embryo quality measures were generally lower than in the presence of FSH (Tables 2 and 3). In the absence of FSH, blastocyst development with pro-mature BMP15 was also significantly higher than the control on day 7 (P<0.05). The trend for improved blastocyst development with mature BMP15 in the presence of FSH was not evident in the absence of FSH. Mature GDF9 without FSH did not improve embryo development or quality.

### Effect of pro-mature BMP15, mature BMP15 and mature GDF9 on COC glucose consumption and lactate production

**Figure 3:**
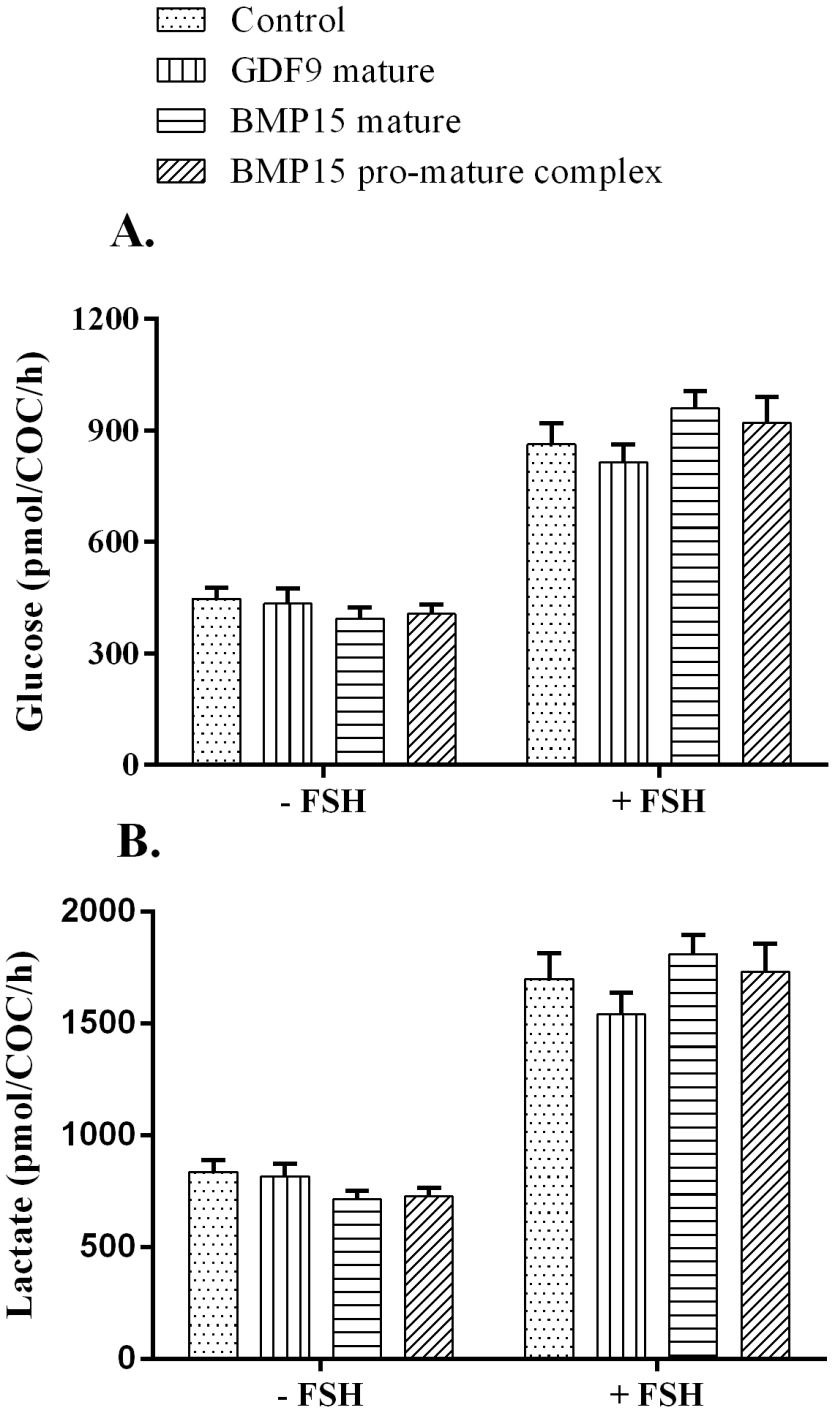
COC glucose consumption and lactate production. Spent IVM medium were analysed for glucose and lactate levels post 23(mature BMP15 and pro-mature BMP15) or mature GDF9 at 100 ng/ml, in the absence or presence of FSH. **A.** Glucose uptake. **B.** Lactate production. Bars represent the mean ± SEM. Data were derived from 12 independent replicates for each treatment.

None of the BMP15 or GDF9 proteins tested altered COC glucose consumption or lactate production, regardless of FSH treatment (P>0.05). As expected, there was an increase in COC glucose uptake and lactate production (almost 2-fold) in response to FSH, across all BMP15/GDF9 treatment groups and controls (Fig. 3).

### Effect of pro-mature BMP15, mature BMP15 and mature GDF9 on oocyte FAD and NAD(P)H autofluorescence

**Figure 4:**
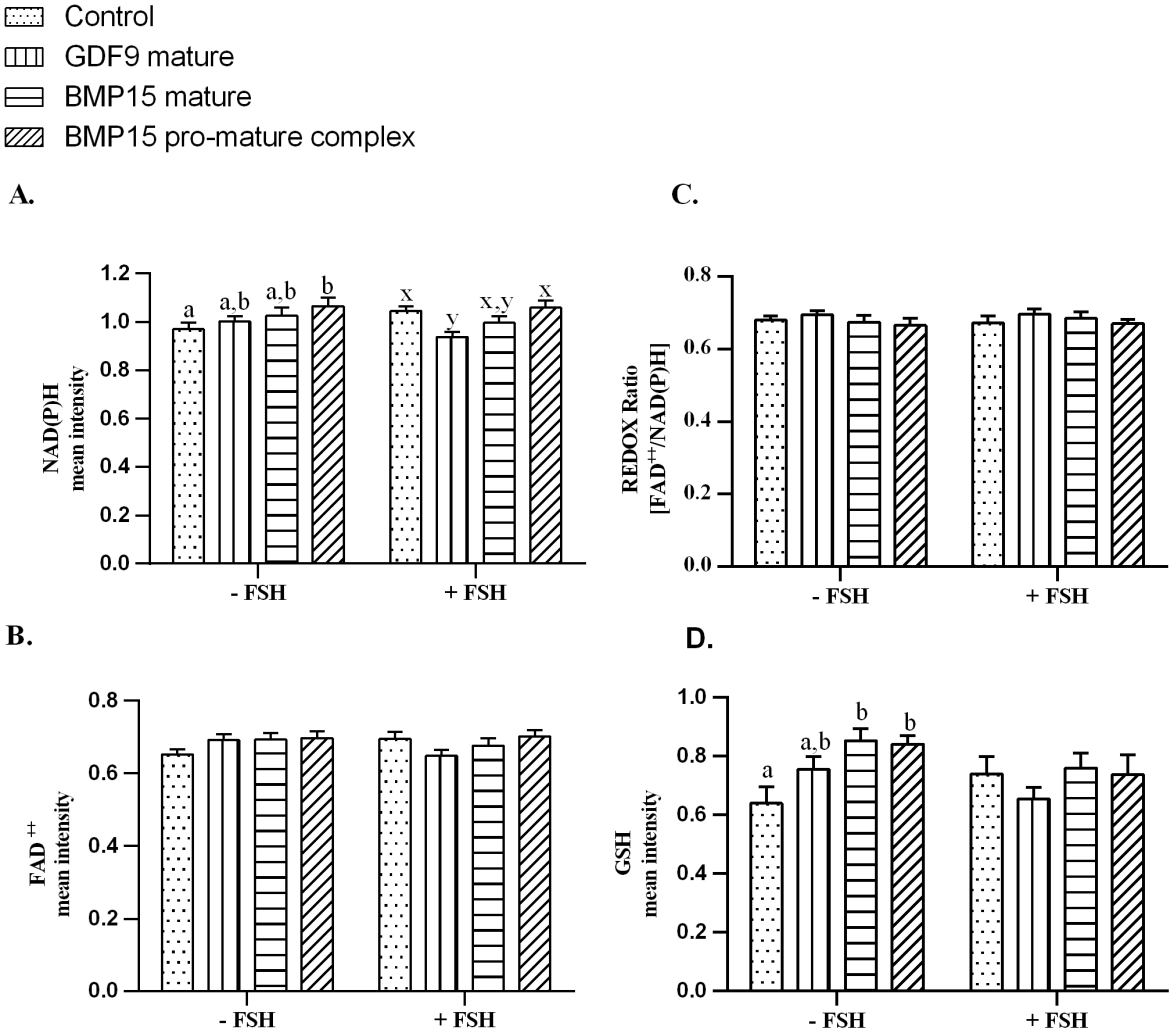
Quantification of intra-oocyte NAD(P)H, FAD, REDOX ratio and GSH. Effect of treatment of intact COCs with different forms of BMP15 (mature BMP15 and pro-mature BMP15) or mature GDF9 at 100 ng/ml, +/− FSH on intra-oocytemetabolic activity. **A.** Autofluorescence of NAD(P)H. **B.** Autofluorescence of FAD. **C.** REDOX ratio (FAD/NAD(P)H). **D.** GSH levels. Bars represent the mean ± SEM. Data were derived from 4 independent replicates for autofluorescence on intra-oocyte NAD(P)H, FAD, and REDOX ratio and 3 independent replicates for GSH levels. Columns with different superscripts are significantly different (P<0.05); ^a,b^minus FSH, ^x–y^plus FSH.

**Figure 5:**
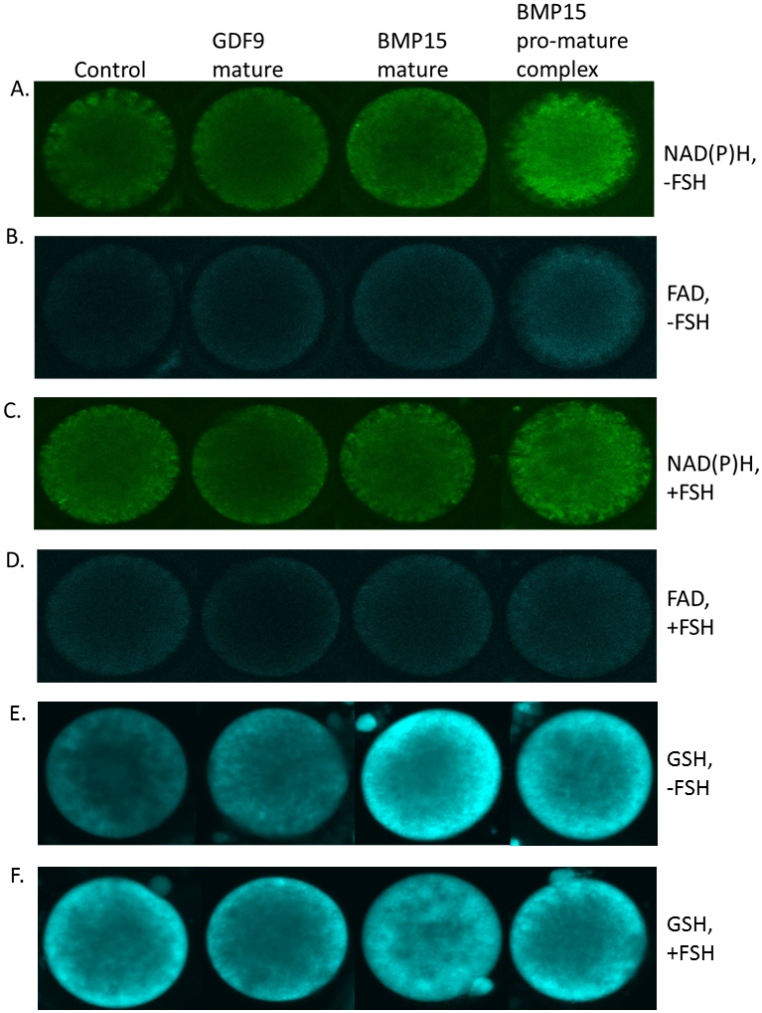
Representative micrographs of intra-oocyte NAD(P)H (A, C) and FAD (B, D) autofluorescence and GSH fluorescence (E, F), after treatment with different forms of BMP15 (mature BMP15 and pro-mature BMP15) or mature GDF9 at 100 ng/ml, in the absence (A, B, E) or presence (C, D, F) of FSH.

Across all treatments, FSH had no effect on intra-oocyte NAD(P)H, FAD, or the REDOX ratio, as assessed by confocal microscopy and analysed by 2-way ANOVA (main effect FSH; P>0.05). In the absence of FSH, there was significantly higher autofluorescence of NAD(P)H in the oocytes treated with pro-mature BMP15 compared to controls (P<0.05, Fig. 4A, Fig. 5A). However, in the presence of FSH, the NAD(P)H level in controls increased to the same level as pro-mature BMP15. There was a significantly lower level of NAD(P)H autofluorescence in oocytes matured in the presence of GDF9 compared to control and pro-mature BMP15 groups in the presence of FSH (P<0.05, Fig. 4A, Fig. 5C). No increase in autofluorescence of FAD (Fig. 4B, Fig. 5B, 5D) or change in REDOX ratio (Fig. 4C) in any treatment groups was observed compared to controls.

### Effect of pro-mature BMP15, mature BMP15 and mature GDF9 on oocyte GSH

Since a significant increase in NAD(P)H levels in oocytes treated with pro-mature BMP15 (-FSH) was detected, we then examined if this was associated with an increase in oocyte GSH, on the basis that if NADPH increased, then the capacity to regenerate reduced GSH was a likely consequence. In the absence of FSH, both forms of BMP15 significantly increased oocyte GSH levels compared to the controls (Fig. 4D, Fig. 5E). Mature GDF9 produced an intermediate level of GSH. However, in the presence of FSH, the GSH level of controls was comparable with all other treatments (Fig. 4D, Fig. 5F). There was no overall effect of FSH on oocyte GSH levels (main effect FSH; P>0.05).

## Discussion

In this present study, we compared the effects of different forms of BMP15 (pro-mature and mature) and the mature form of GDF9 during IVM on bovine embryo development. The pro-mature form of BMP15 was the only form of OSF that significantly increased subsequent embryo development relative to the control. Mature domain GDF9 was ineffective and mature domain BMP15 lead to a moderate, albeit non-significant, improvement in embryo yield. We have also produced pro-mature human GDF9. It is known that this form of human GDF9 is naturally latent [46], [47] and consistent with this, we have recently shown that this protein has no effect on bovine COCs [38]. Moreover, the utilization of this protein is problematic in culture, as we have found that it has a very high binding affinity for plastic culture-ware (Mottershead DG, unpublished data) and we have not yet determined a suitable culture environment.

Oocyte-secreted growth factors have been studied extensively either in their native form (i.e. as secreted by oocytes) or as recombinant growth factors [1]. It is now a well-known concept that co-culture of denuded oocytes (as a source of native exogenous OSFs) with COCs improves developmental competence of mouse [39], pig [48], cattle [35], [37], [38], [49] and goat [50] IVM oocytes. However, a full characterisation of the forms and indeed the complete identities of native OSFs has not been undertaken. Previous results showed that addition of either non-purified BMP15 or GDF9 (containing pro-mature complexes) improved cattle blastocyst development, and pro-mature mouse GDF9 improved mouse fetal yield [35]–[37]. However, so far, there is no published report of any significant improvements after addition of the only commercially available mammalian cell expressed mature regions of GDF9 or BMP15. Our previous findings indicated that addition of the mature regions of GDF9 or BMP15 (R&D Systems) did not improve the developmental competence of mouse IVM oocytes [39].

Although the form of GDF9 and BMP15 which are produced by the oocyte and released into the somatic cell compartment of the follicle remains elusive, it is known that both the pro-region and mature region play important roles in the function of these proteins. The mature domain of TGF-β superfamily proteins is the bioactive receptor binding region, and while the mature domain is complexed with the pro-region after processing to form the pro-mature complex, this form can either be latent or active depending on the particular superfamily member [51]–[53]. In the case of human GDF9, the association of the pro-region with its mature region confers latency [46], [47]. Further, the pro-region has important functions in protein folding, formation of the disulfide bonds and regulation of bioactivity [51], [54]. Indeed the pro-mature complex of BMP15 was highly effective at enhancing oocyte quality during IVM [14], [38]. It seems plausible that the pro-domain of BMP15 may interact with the extracellular matrix of cumulus cells during maturation and facilitate presentation of the mature domain to cumulus cell receptors. In support of this concept, it has been shown that the GDF9 pro-mature complex binds strongly to heparin sepharose, suggesting that heparan sulfate proteoglycans on cumulus cells may act as co-receptors mediating oocyte secreted factor signalling [34]. Collectively, these results indicate that the pro-domains of GDF9 and BMP15 exhibit important interactions with ovarian somatic cells, and may explain why the isolated mature regions of GDF9 and BMP15 appear to have little effect on oocyte developmental competence.

*In vitro* matured COCs have aberrant expression of genes and proteins compared to COCs matured in vivo [55]–[57]. Notably, mouse COCs express processed BMP15 protein during *in vivo* oocyte maturation, but not during IVM [30]. From our data, it is clear that BMP15 improved bovine embryo development compared to GDF9. However, GDF9 may have the capability to improve oocyte quality during IVM if it were in a different form; e.g. as a pro-mature complex or if bovine GDF9 were used instead of mouse GDF9. Different species origins of recombinant OSFs have different effects on granulosa cell sterol biosynthesis and bioactivity [58], [59]. Interestingly, mouse GDF9 in its mature form is significantly more bioactive on bovine granulosa cells compared to BMP15 (both forms; current study). Hence, the lack of effect of mouse GDF9 on oocyte competence was not due to lack of bioactivity of this preparation on bovine cells.

The improvement of embryo development after addition of pro-mature BMP15 was accompanied by an increase in oocyte autofluorescence of NAD(P)H. However, there was no increase in oocyte FAD or change in REDOX status. These results differ slightly from our previous published data which showed that the addition of pro-mature BMP15 increased not only NAD(P)H but also increased FAD levels [14]. Unfortunately, we cannot distinguish between autofluorescence from NADPH derived from the pentose phosphate pathway (PPP) or isocitrate dehydrogenase activity, and autofluorescence from NADH, which is a product of several metabolic pathways including glycolysis, and the tricarboxylic acid (TCA cycle). If the increasing level of NAD(P)H in the oocyte after treatment with pro-mature BMP15 is in part due to increased NADPH, then this may also relate to the higher levels of GSH in oocytes, as NADPH is required for reduction of oxidised glutathione via the enzyme glutathione reductase. Glutathione is an antioxidant molecule in the cell and acts as a defence mechanism in oxidative stress [60], [61]. Reactive oxygen species (ROS), such as superoxide anion, react with the thiol of GSH and oxidise glutathione (GSSG) [60]. As a reducing agent, NADPH aids in the conversion of GSSG into GSH. Raising the level of GSH during IVM is well documented to improve oocyte quality during bovine oocyte maturation [62], [63]. Interestingly, even though there was no increase in NAD(P)H levels in COCs matured in the presence of mature BMP15, the level of GSH was significantly increased compared to control. This is perhaps due to lower levels of ROS production in this group, something that could be confirmed in future experiments.

The aim of testing the medium in the absence of FSH was to observe the function of exogenous growth factors on IVM oocytes without any influence of metabolic activators. Cumulus cell function during IVM is profoundly altered by FSH treatment, including cumulus cell energy metabolism [64]. The present study shows that in the absence or presence of FSH, the addition of pro-mature BMP15 to IVM increases significantly embryo development on day 7 and yielded more blastocysts on day 8 compared to any other group. As expected, FSH also improved IVM outcomes. However, in the presence of FSH, the influence of recombinant OSFs on NAD(P)H and GSH levels were diminished. These results are similar with the previous reports that showed FSH can mask the influence of OSFs on cumulus cell metabolism [14], [65].

It is well established that during IVM, COCs utilise glucose as the principal energy source and glucose consumption increases linearly with oocyte maturation [66]. In this present study, glucose uptake and lactate production were measured in the presence or absence of FSH. It has been shown that in the absence of alteration of metabolism by FSH, the influence of OSFs on glycolytic activity leads to the activation of oxidative phosphorylation in mitochondria to produce ATP, rather than lactate production [13], [14]. Our collaborators have shown that addition of mature BMP15 increases glucose uptake but has no effect on lactate production by Nellore (*Bos indicus*) bovine COCs, under slightly different culture conditions [67]. We did not observe any increases in glucose uptake in any OSF treatment group, including with mature BMP15, in the presence or absence of FSH. This result is supported by our previous study [14].

In conclusion, we found that the form of the oocyte-secreted growth factors GDF9 and BMP15 is an important determinant of their function during oocyte IVM, and that it affects subsequent embryo development. Only the pro-mature form of BMP15 added in IVM medium significantly improved oocyte developmental competence, and importantly from a practical perspective, there was no evidence that the only commercially available mammalian forms of GDF9 and BMP15 could increase cattle embryo production. Improvement of bovine IVM oocyte competence, may be, in part, due to an increase in oocyte NAD(P)H and GSH.
